# Recombinant *Echinococcus granulosus* myophilin alleviates airway inflammation in mice with ovalbumin-induced allergic asthma via the gut microbiota-metabolite-immune axis

**DOI:** 10.1186/s13071-026-07444-8

**Published:** 2026-05-19

**Authors:** Zhichao Zhou, Leiji Fu, Jianwen Wu, Rou Wen, Zexin Dang, Junyou Wu, Xiaomin Zhang, Sijia Bao, Wenxuan Li, Xiaoping Gao, Mei Yin, Jiaqing Zhao

**Affiliations:** 1 School of Basic Medicine, Ningxia Medical University, Yinchuan, China; 2 School of the First Clinical Medical, Ningxia Medical University, Yinchuan, China; 3 Ningxia Key Laboratory for Prevention and Control of Common Infectious Diseases, Ningxia Medical University, Yinchuan, China; 4 Department of Respiratory and Critical Care Medicine, Hospital of Cardiovascular and Cerebrovascular Diseases, General Hospital of Ningxia Medical University, Yinchuan, China; 5 Department of Otolaryngology Head and Neck Surgery, General Hospital of Ningxia Medical University, Yinchuan, China; 6 Ningxia Institute of Medical Science, Ningxia Medical University, Yinchuan, China

**Keywords:** Allergic asthma, Intestinal flora, Metabolites, Immune regulation, r*Eg*.myophilin

## Abstract

**Background:**

Parasitic infections or their secreted components exhibit therapeutic effects against certain allergic diseases. Allergic asthma is a chronic inflammatory airway disease with potentially severe symptoms and increasing prevalence worldwide. Recombinant *Echinococcus granulosus* myophilin (r*Eg*.myophilin) induces a Th1 immune response in mouse spleens; however, the effects and mechanisms of r*Eg*.myophilin in allergic asthma remain unclear.

**Methods:**

We investigated the anti-inflammatory effects of r*Eg*.myophilin on airway inflammation in mice with ovalbumin (OVA)-induced allergic asthma through histopathology, flow cytometry, and enzyme-linked immunosorbent assay. 16S rRNA sequencing and non-targeted metabolomics of mouse fecal samples and correlation analyses of microbiota, metabolites, and inflammatory indicators were used to explore the mechanistic role of r*Eg*.myophilin in allergic asthma.

**Results:**

r*Eg*.myophilin significantly ameliorated OVA-induced allergic airway inflammation. Pathological findings revealed a marked reduction in lung inflammatory cell infiltration, collagen deposition, and mucus secretion. r*Eg*.myophilin also corrected the imbalance in Th1/Th2 cell ratios in lung tissues and reduced the abundance of *Tenericutes* and *Candidatus_Arthromitus*. Among the 19 metabolites with significant differences among Con, OVA, and OVA+r*Eg*.myophilin groups, those linked to linoleic acid metabolism, indicating that r*Eg*.myophilin may act through the linoleic acid metabolic pathway to alleviate allergic asthma. Spearman's correlation analysis revealed positive/negative correlations between several differential metabolites, differential microbiota, and immune indicators.

**Conclusions:**

r*Eg*.myophilin alleviates OVA-induced allergic asthma in mice by modulating interactions among intestinal microbiota, metabolites, and immune cells. This research provides theoretical insights and novel biological targets for the prevention and treatment of allergic asthma.

**Graphical abstract:**

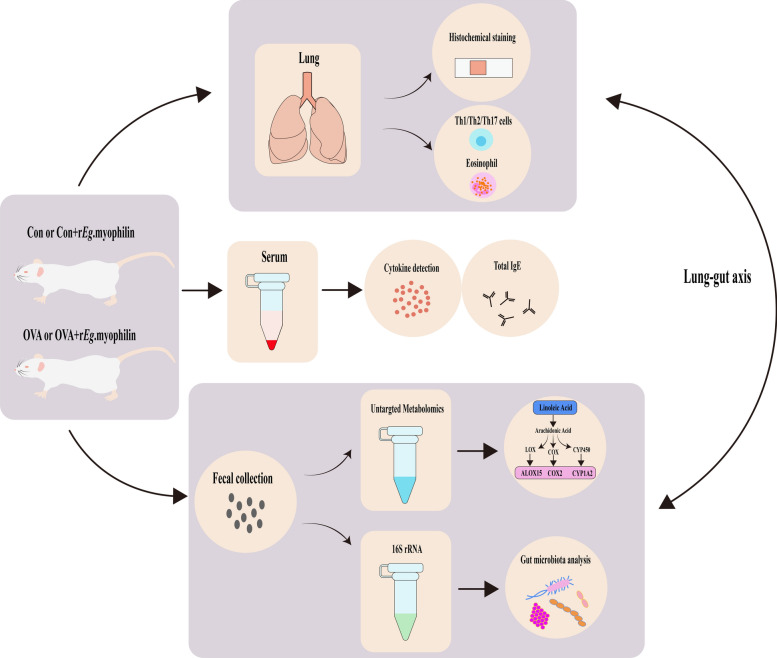

**Supplementary Information:**

The online version contains supplementary material available at 10.1186/s13071-026-07444-8.

## Background

Asthma is a chronic inflammatory airway disease characterized by persistent or intermittent airway hyperreactivity, reversible airway obstruction, and airway remodeling. Asthma incidence is rapidly increasing, impacting over 300 million people worldwide, and is closely linked to allergen exposure [1]. People with asthma can experience severe symptoms such as wheezing, coughing, and breathing difficulties. The pathogenesis of classical allergic asthma primarily involves an imbalance in the CD4^+^ T helper 1 (Th1)/Th2 cell ratio and overproduction of Th2 cell-associated cytokines, such as interleukin (IL)−4, IL-5, and IL-13 [2]. Imbalance in CD4^+^ Th17 also contributes to allergic asthma's pathogenesis [3]. Current treatments for allergic asthma primarily rely on traditional inhaled glucocorticosteroids and β2-agonists. Despite the recent emergence of targeted immunotherapy methods, such as omalizumab (anti-immunoglobulin E [IgE] biologics), increasing drug resistance and application limitations pose ongoing challenges [4, 5]. Therefore, exploring novel, sensitive, effective treatment modalities remains a focus of allergic asthma research.

According to the hygiene hypothesis, many allergies and antoimmune diseases are associated with reduced exposure to parasites [6], prompting increasing interest in the therapeutic potential of parasitic infections or specific worm secretions and components. For instance, a protein secreted by the mouse helminth parasite can inhibit type 2 allergic immune responses by binding to and interfering with IL-33 release [7]. Echinococcosis is a zoonotic disease caused by infection with *Echinococcus granulosus*. Infection with *E. granulosus* can significantly reduce the proportion of eosinophils in alveolar lavage fluid in mice [8]. Additionally, *E. granulosus* cystic fluid significantly attenuates lung histopathology in mice with ovalbumin (OVA)-induced allergic asthma [9]. *Echinococcus granulosus* myophilin is expressed in the subperitoneal parenchyma of protoscolex and adult parasite suckers; this protein regulates host tissue invasion and helminth motility by modulating smooth muscle contraction [10]. Recombinant *E. granulosus* myophilin (r*Eg*.myophilin), purified by in vitro expression through genetic engineering technology, is soluble and antigenic, and it can generate specific responses in organisms, inducing splenocyte proliferation and Th1 responses in mice [11, 12]. Compared with adult parasite infections, r*Eg*. myophilin avoids multiple antigen stimulation and confounding factors [13]. Despite promising evidence that infection with *E. granulosus* and oral ingestion of *E. granulosus* cystic fluid can alleviate OVA-induced asthma, the potential for adverse effects is unknown. Therefore, exploring the safe and effective components of *E. granulosus* and determining their mechanism of action are vital for validating its therapeutic potential.

Growing evidence suggests that the intestinal microbiota is instrumental in asthma development and progression [14]. Untargeted metabolomics analyses of the gut microbiome support this hypothesis, demonstrating an association between gut microbiota and metabolites with wheeze frequency in children with asthma [15]. Moreover, mice infected with *E. granulosus* exhibit significant differences in intestinal microbial communities, providing theoretical insights into host-parasite interactions and the therapeutic mechanism of *E. granulosus* [16].

Therefore, in this study, we investigate the therapeutic efficacy and mechanism of action of r*Eg*.myophilin in allergic asthma. Specifically, we explored the effects of r*Eg*.myophilin infection on inflammatory indicators and intestinal microbiota and metabolites in an OVA-induced mouse model of allergic airway inflammation. We also elucidate the relationship among microbiota, metabolic pathways, and immune regulation to provide new insights into asthma treatment strategies.

## Methods

### Ethical statement and experimental design

This study strictly adhered to the Guidelines for the Care and Use of Laboratory Animals of the Ministry of Science and Technology of the People's Republic of China. All animal experiments were approved by the Medical Ethics Committee of General Hospital of Ningxia Medical University (license no. KYLL-2021-655) and conducted in strict accordance with these guidelines. The r*Eg*.myophilin used in this study was purified using genetic engineering techniques, and the solution was subsequently filtered and decontaminated through a 0.22-μm filter before administration to the mice [12].

Female, pathogen-free mice (6–8 years old, weighing 20 ± 2 g) were purchased from Beijing Weitong Lihua Laboratory Technology Co., Ltd. (Qualification Certificate: SYXK Ning 2020-0001). Before starting the experiment, mice were housed in a specific-pathogen-free animal house with access to drinking water and autoclaved food.

Female specific- pathogen-free BalB/C mice were randomly divided into four groups: control group (Con), control+r*Eg*.myophilin group (Con+r*Eg*.myo), OVA-induced asthma model group (OVA), and OVA-induced asthma model+r*Eg*.myophilin group (OVA+r*Eg*.myo). For the asthma model groups (OVA and OVA+r*Eg*.myo), mice were injected intraperitoneally with 20 μg of a mixture containing OVA (A5503-1G, Sigma-Aldrich, Germany) and aluminum hydroxide adjuvant (77161, Thermo Fisher Scientific, USA) per mouse on days 0, 7, and 14. For the control groups (Con and Con+r*Eg*.myo), mice received an equal amount of phosphate-buffered saline (PBS) (G4202, Servicebio, China) via the same route. For the intervention groups (Con+r*Eg*.myo and OVA+r*Eg*.myo), mice received 20 μg of r*Eg*.myophilin subcutaneously on days −1, 6, and 13, with all other mice receiving an equal volume of PBS. Then, from day 21 to 27, mice in the allergic asthma model groups (OVA and OVA+r*Eg*.myo) were administered 100 μg of OVA intranasally daily, with all other mice receiving an equal volume of PBS buffer. All mice were monitored daily for appetite, body temperature, and activity levels. For subsequent analyses, seven mice per group were used for flow cytometry, lung tissue pathology assessment, and serum testing, and six mice per group were used for fecal microbiota and metabolite analyses.

### Chemical staining and immunohistochemical (IHC) analysis of lung tissue

The lower lobe of the left lung from each mouse was collected, inflated, and fixated overnight in 4% paraformaldehyde (G1101, Servicebio, China). Subsequently, the tissues were embedded in paraffin, sectioned, and stained with hematoxylin and eosin (H&E) for airway inflammation analysis, periodic acid-Schiff (PAS) for mucus production analysis, myeloperoxidase (MPO) for inflammatory cell infiltration analysis, and Masson’s trichrome stain for collagen deposition visualization. Quantitative analysis was performed using Fiji software (version 2.16.0).

### Preparation of single-cell suspensions of lung tissue

After the mice were killed, lung tissues were collected, dissected, and immersed in 1 mg/mL of type I collagenase solution (C8140, Solarbio, China) with shaking at 220 rpm for 1 h. The sheared lung tissues were then incubated in a pre-cooled complete culture medium (1640 medium containing 5% fetal bovine serum) and filtered through a 300-mesh nylon filter. The resulting cell suspension was treated with an erythrocyte lysis solution for red blood cell lysis (R1010, Solarbio, China). After washing twice with PBS, the cells were counted for flow cytometry analysis.

### Flow cytometry

Single-cell suspensions of mouse lung tissues were prepared as described above. The following staining labeling protocols were used for flow cytometry analysis: APC-Cy7-anti-mouse-CD45 (557659, 30-F11, BD, USA), FITC-anti-mouse-CD11b (101206, M1/70, BioLegend, USA), PE-anti-mouse-Gr-1 (108408, RB6-8C5, Biolegend, USA), APC-anti-mouse-CD170 (155,508, S17007L, BioLegend, USA), FITC-anti-mouse-CD3e (100306, 145-2C11, BioLegend, USA), APC-Cy7-anti-mouse-CD3 (100222, 17A2, BioLegend, USA), FITC-anti-mouse-CD4 (100510, RM4-5, BioLegend, USA), APC-anti-mouse-CD4 (100412, GK1.5, BioLegend, USA), APC-anti-mouse-IFN-γ (163513, W18272D, BioLegend, USA), PE -anti-mouse- IL-4 (504104, 11B11, BioLegend, USA), and Bv421-anti-mouse-IL-17A (506926, TC11-18H10.1, BioLegend, USA). Th1/2/17 cells were treated with a cell stimulant for 4 h prior to subsequent experiments (Cell Activation Cocktail: contains PMA, ionomycin, and Brefeldin A, 423304, BioLegend, USA). For flow cytometric detection, intracellular cytokines were fixed and permeabilized using fix/perm buffer (88–8824-00, Thermo Fisher Scientific, USA). Cell viability was detected using the Zombie Aqua™ Fixable Viability Kit (423101, BioLegend, USA). All stained cell samples were analyzed using a BD FACSCelesta flow cytometer (BD Biosciences, San Jose, CA, USA). Analysis was performed using FlowJo software (version10.10.0).

### Total IgE and cytokine assay in mouse serum

Total IgE levels in mouse serum were measured by enzyme-linked immunosorbent assay according to the manufacturer’s instructions (88–50460-22, Thermo Fisher Scientific, USA). Mouse serum levels of the cytokines IL-4, IL-6, IL-17A, IL-10, and tumor necrosis factor (TNF) were assayed using the Cytometric Bead Array (CBA) Mouse Th1/Th2/Th17 Cytokine Kit (560485, BD, USA) according to the manufacturer’s protocol. All samples were analyzed using a BD Accuri C6 flow cytometer and FCAP Array v3.0 software (BD, USA).

### 16S rRNA sequencing analysis of gut microbiota in mice

Fecal samples from all four groups of mice were collected and sequenced for the V3–V4 region of 16S rRNA on Agilent 2100 Bioanalyzer (Agilent Technologies, Santa Clara, CA, USA) and Illumina (Kapa Biosciences, Woburn, MA, USA) platforms. Sequencing data processing involved removing splices and barcode sequences based on barcode information. RawData primer sequences and balanced base sequences were removed using Cutadapt software(version 1.9). Paired-end reads were merged into a single, longer tag based on the overlap region using FLASH (version 1.2.8). Subsequently, the DADA2 pipeline through the QILME “dada2 denoise-paired” plugin was used for length filtering and denoising to obtain amplicon sequence variant feature and abundance tables. Species annotation was performed using the SILVA and NT-16S databases based on amplicon sequence variant sequence file. The abundance of each species in each sample was determined based on the amplicon sequence variant abundance table. The confidence threshold for annotation was set to 0.7.

### Mass spectrometric analysis of non-targeted metabolomics in mouse fecal samples

Collected fecal samples were melted, and the metabolites were extracted from 20-µl aliquots using pre-cooled 50% methanol buffer (67-56-1, Honeywell, USA). Quality control samples were mixed with all other samples and analyzed in both positive and negative ion modes using a TripleTOF 5600 Plus high-resolution tandem mass spectrometer (SCIEX, Warrington, UK); liquid chromatography-mass spectrometry data were preprocessed using XCMS software. Raw data files were converted to mzXML format and processed using the XCMS, camera, and metaX toolboxes in R software. Metabolites were annotated using the Kyoto Encyclopedia of Genes and Genomes (KEGG) and Human Metabolome Database. Finally, supervised Partial Least Squares Discriminant Analysis (PLS‐DA) was performed to identify specific differences between groups.

### Quantitative real-time PCR

Mouse lung tissues were thoroughly ground in TrizoL Reagent (15596018CN, Thermo Fisher Scientific, USA), followed by RNA extraction and reverse transcription of RNA to cDNA according to the instructions of the reverse transcriptase reagent manufacturer (RR036A, TaKaRa, Japan), and quantitative PCR using qPCR reagent (DBI-2043, DBI Bioscience, Germany). Relative gene expression was calculated using 2-ΔΔCt.

### Western blotting

Total proteins from mouse lung tissues were extracted and quantified using the Total Tissue Protein Extraction Kit and the BCA Protein Quantification Kit following the reagent manufacturers' instructions. Proteins were subsequently separated using sodium dodecyl sulfate-polyacrylamide gel electrophoresis and transferred to polyvinylidene fluoride membranes, closed with 5% skimmed milk powder, then incubated overnight with the following antibodies—ALOX15 (ab244205, EPR22138, Abcam, UK), COX2 (ab179800, EPR12012, Abcam, UK), CYP1A2 (ab314666, EPR28416-31, Abcam, UK), and β-actin (Cell signaling Technology, USA)—before a final incubation and subsequent detection using goat anti-rabbit secondary antibody (Cell signaling Technology, USA). Grayscale values were quantified using Fiji (ImageJ, v 2.16.0) software.

### Statistical analysis

Data for each group were expressed as the mean ± standard deviation. One-way analysis of variance was used to compare multiple groups of data with GraphPad-Prism (version 8.0) statistical graphing software, and *p* < 0.05 was considered statistically significant. Correlation analysis was performed using Spearman's analysis, and *p* < 0.05 was considered statistically significant.

## Results

### r*Eg*.myophilin effectively alleviates allergic airway inflammation in mice

We evaluated the effects of OVA-induced allergic asthma in a mouse model and r*Eg*.myophilin intervention using H&E, Masson’s trichrome, MPO, and PAS staining on lung tissue samples from the four mouse groups.

**Fig. 1:**
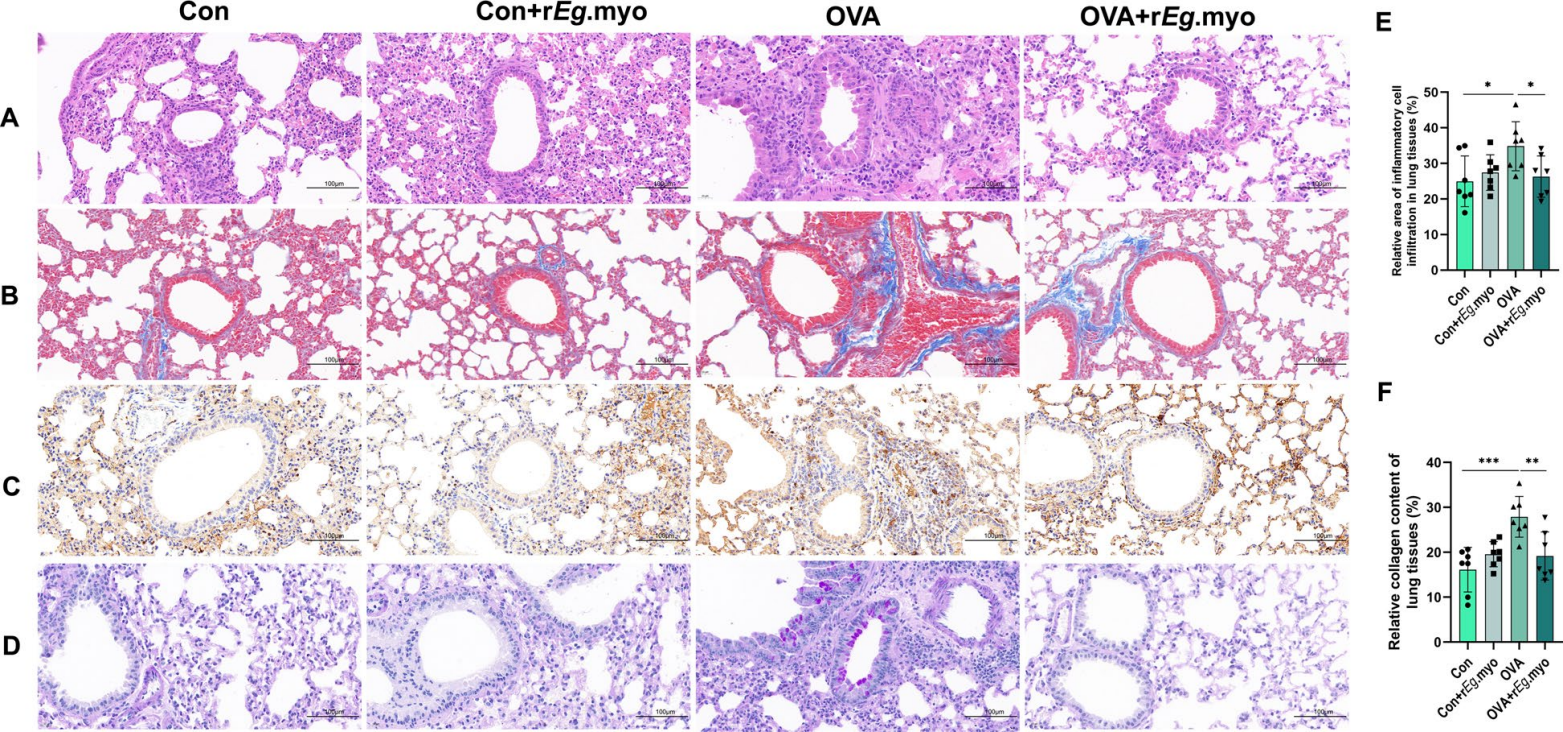
Effects of r*Eg*.myophilin intervention on airway inflammation, collagen deposition, mucus secretion, and myeloperoxidase production in mice. **A** Hematoxylin and eosin (H&E) staining; **B** Masson’s trichome staining; **C** myeloperoxidase staining; **D** periodic acid-Schiff (PAS) staining; **E** relative infiltration area of inflammatory cells in H&E-stained lung tissues; **F** relative collagen content in Masson’s trichome-stained lung tissues (magnification: 40 ×; scale bar: 100 μm); *n* = 7; * *p* < 0.05, ** *p* < 0.01, *** *p* < 0.001

**Fig. 2:**
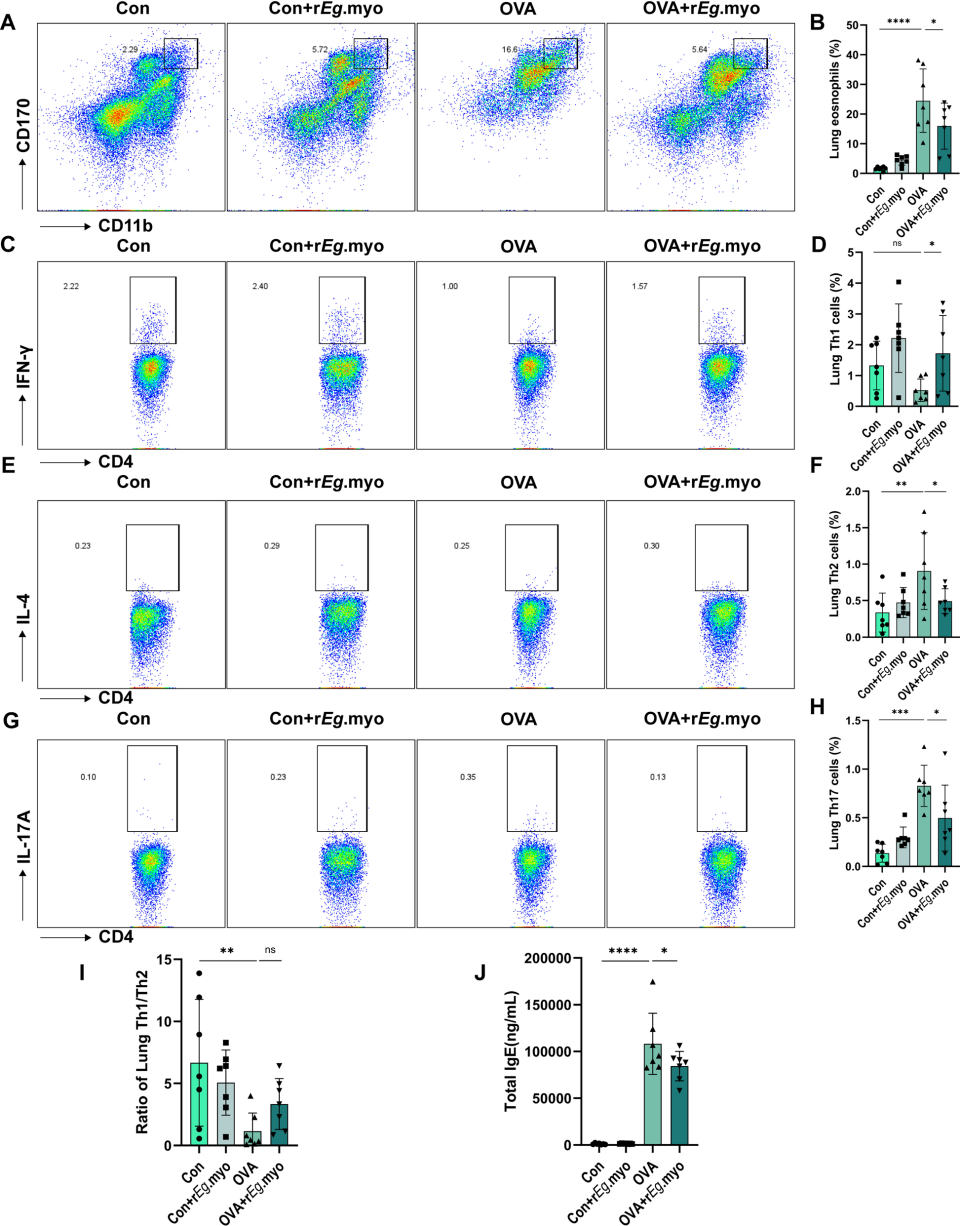
Effects of r*Eg*.myophilin intervention on the percentages of eosinophils and T helper (Th)1, Th2, and Th17 cells in the lungs and total immunoglobulin E (IgE) levels in the serum of mice. **A**–**B** Eosinophils; **C**–**D** Th1 cells; **E**–**F** Th2 cells; **G**–**H** Th17 cells; **I** ratio of Th1/Th2 cells; **J** serum total IgE levels; *n* = 7; * *p* < 0.05, ** *p* < 0.01, *** *p* < 0.001, **** *p* < 0.0001

Compared with the Con group, the OVA group showed significant infiltration of inflammatory cells into airway tissues (Fig. 1A and E), extensive collagen fiber deposition (Fig. 1B and F), a clear area of myeloperoxidase positivity (Fig. 1C), marked goblet cell proliferation, and increased mucus secretion (Fig. 1D). Compared with the OVA group, the OVA+r*Eg*.myo group showed reduced inflammatory cell infiltration in the airway tissues (Fig. 1A and E), collagen deposition (Fig. 1B and F), intensity of medullary peroxidase-positive areas (Fig. 1C), and reduced goblet cell proliferation and mucus secretion (Fig. 1D). Flow cytometry analysis of eosinophils in the lungs of mice showed a significantly greater proportion of eosinophils in the OVA group than in the Con group and a significantly lower proportion of eosinophils in the OVA+r*Eg*.myo group than in the OVA group (Fig. 2A–B). The same trends were observed for serum total IgE levels (Fig. 2J). These findings indicated successful induction of the allergic asthma mouse model and demonstrated that r*Eg*.myophilin treatment effectively alleviated allergic airway inflammation.

### r*Eg*.myophilin alleviates Th1/Th2 and Th17 cell imbalance in the lungs of mice with allergic asthma

An imbalanced Th1/Th2 ratio is a classical immunological mechanism in the development of asthma, with Th17 cells more recently implicated in asthma [2, 3]. To assess the immunological mechanism whereby r*Eg*.myophilin alleviates OVA-induced allergic asthma in mice, we examined immune cells in the lungs using flow cytometry. Compared with the Con group, the OVA group showed significantly increased proportions of Th2 and Th17 cells. The proportion of Th1 cells was reduced, indicating a significant reduction in the Th1/Th2 ratio (Fig. 2C–I). Compared with the OVA group, the OVA+r*Eg*.myo group showed a significantly increased proportion of Th1 cells and decreased proportions of Th2 and Th17 cells, indicating an increased Th1/Th2 ratio (Fig. 2C–I). These findings highlight a Th1/Th2 cell imbalance in the OVA-induced allergic asthma model (Fig. 2I), which is modulated by r*Eg*.myophilin treatment in pulmonary immune cells.

### r*Eg*.myophilin regulates cytokine secretion in mice with allergic asthma

**Fig. 3:**
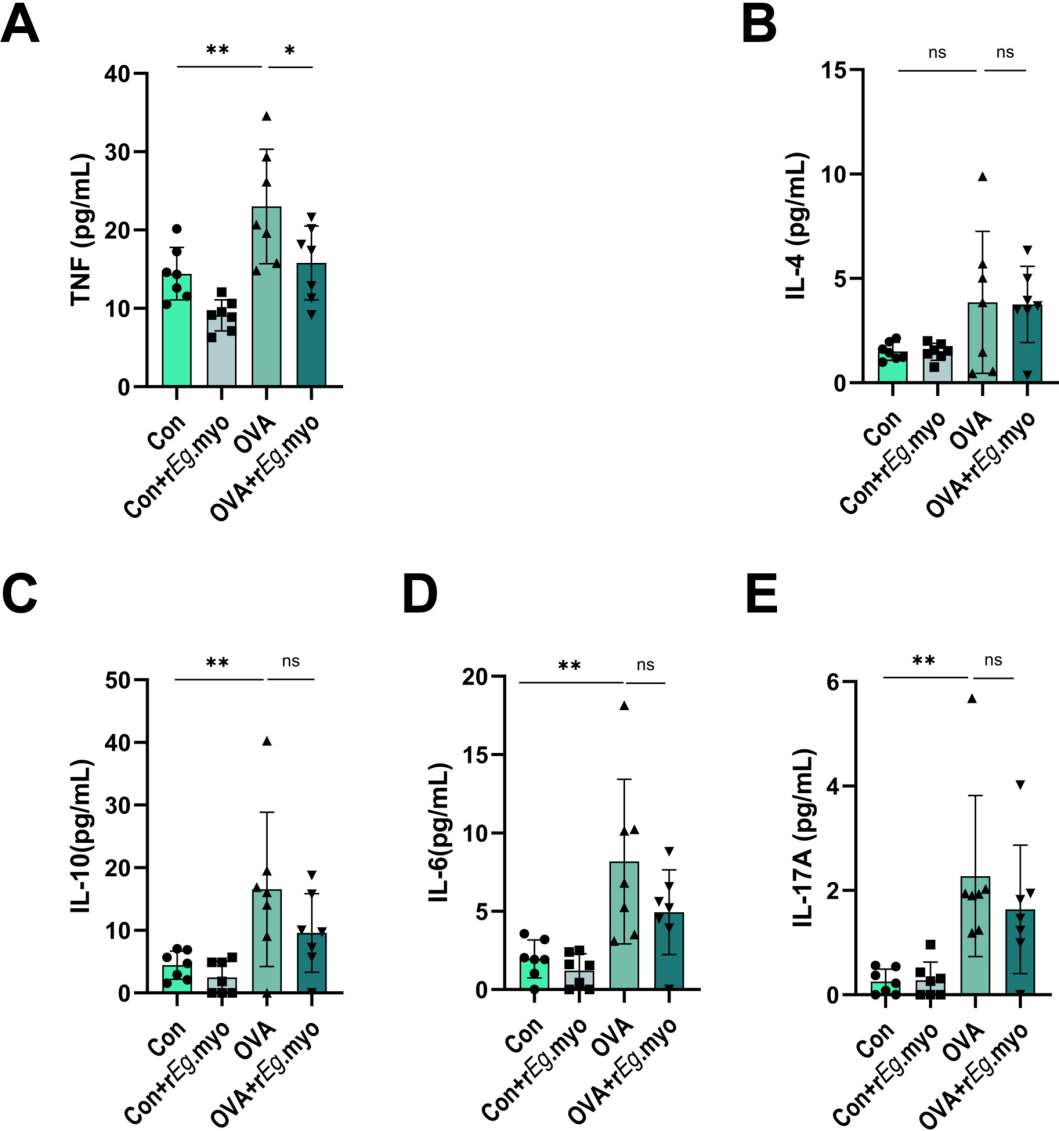
Effects of r*Eg*.myophilin intervention on serum cell cytokine levels in mice. **A** TNF; **B** interleukin (IL)−4; **C** IL-10; **D** IL-6; **E** IL-17A; *n* = 7; * *p* < 0.05, ** *p* < 0.01

Besides immune cells, immune effector molecules known as cytokines are also involved in asthma [17]. Therefore, we also examined cytokine levels in mouse serum. Compared with the Con group, the OVA group showed increased levels of TNF, IL-6, IL-17A, and IL-10. Compared with the OVA group, the OVA+r*Eg*.myo group showed decreased levels of TNF (Fig. 3A). Changes in IL-4, IL-6, IL-10, and IL-17A levels were not significant between groups (Fig. 3B–E). These results reveal multiple cytokine imbalances in the OVA-induced allergic asthma model, which are reversed by r*Eg*.myophilin treatment.

### r*Eg*.myophilin modulates intestinal microbiota dysregulation in mice with allergic asthma

Emerging evidence suggests a bi-directional relationship between gut microbiota and the lung environment [18]. To further investigate the underlying mechanisms of r*Eg*.myophilin in allergic asthma, we sequenced and analyzed the intestinal microbiota of all four groups of mice, then subjected the raw data obtained at the end of sequencing to a series of quality control and data processing steps, as previously described.

**Fig. 4:**
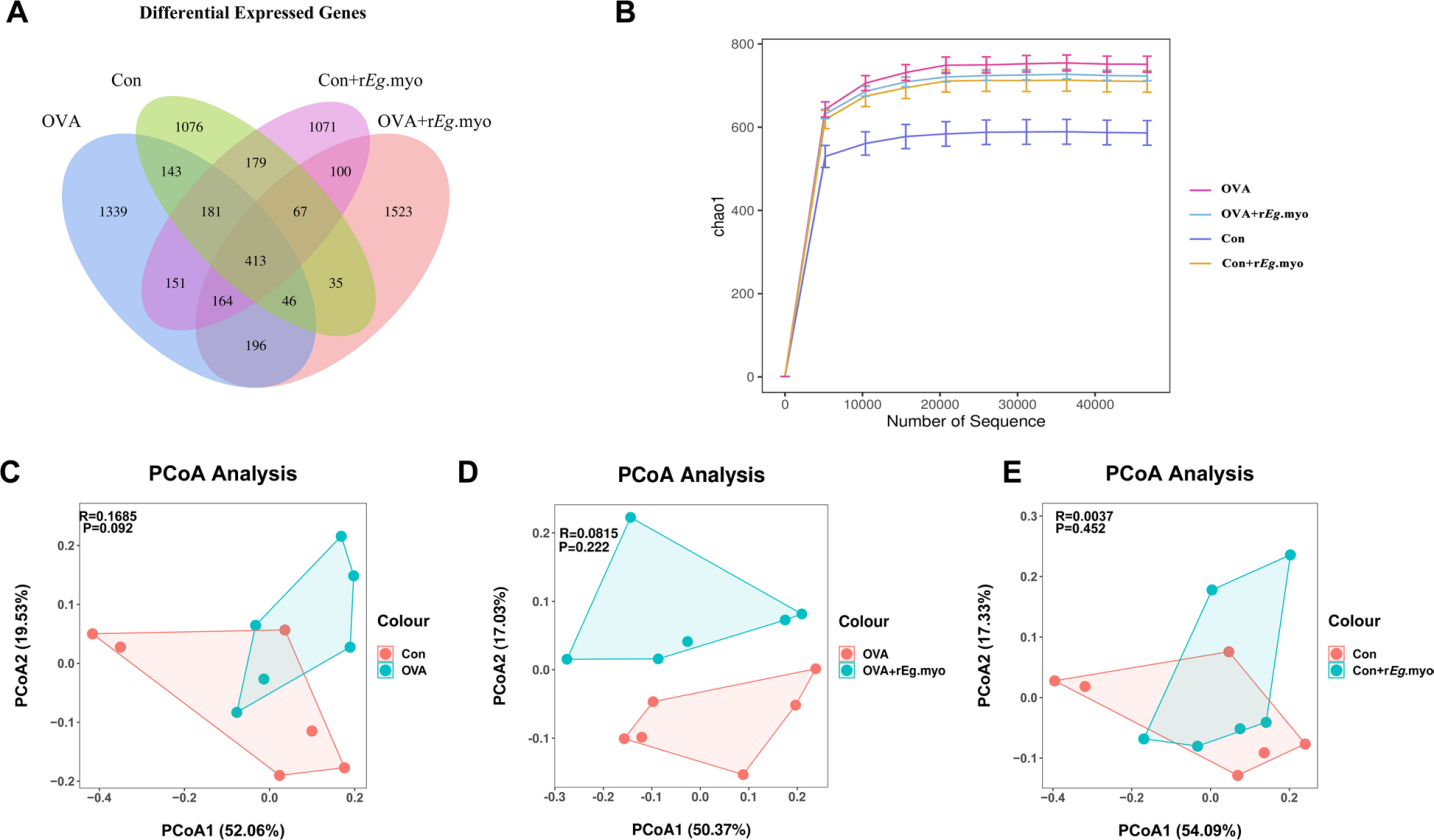
Quality control, α-diversity analysis, and principal coordinate analysis of intestinal microbiota in mouse fecal samples. **A** Feature statistics of the four groups of samples; **B** Chao1 Index curves; **C** control (Con) group vs. OVA-induced asthma model (OVA) group; **D** OVA group vs. OVA+r*Eg*.myo group; **E** Con group vs. Con+r*Eg*.myo group

The OVA+r*Eg*.myo group showed 1523 differential features compared with the other three groups (Fig. 4A). Next, alpha (α) diversity analysis was used to explore species richness and evenness between groups. Dilution curves showed that species richness stabilized when sequencing depth reached 20,000 and was highest in the OVA group and lowest in the Con group (Fig. 4B). Next, we used principal coordinate analysis to visualize differences in microbiota species between groups. Differences in intestinal microbiota were observed between the Con and OVA groups, the OVA and OVA+r*Eg*.myo groups, and the Con and Con+r*Eg*.myo groups (Fig. 4C–E). Thus, the OVA-induced allergic asthma model caused disruptions in the intestinal microbiota of mice, which were modulated by r*Eg*.myophilin treatment.

### r*Eg*.myophilin alters gut microbiota composition and phylum- and genus-level abundances in mice with allergic asthma

**Fig. 5:**
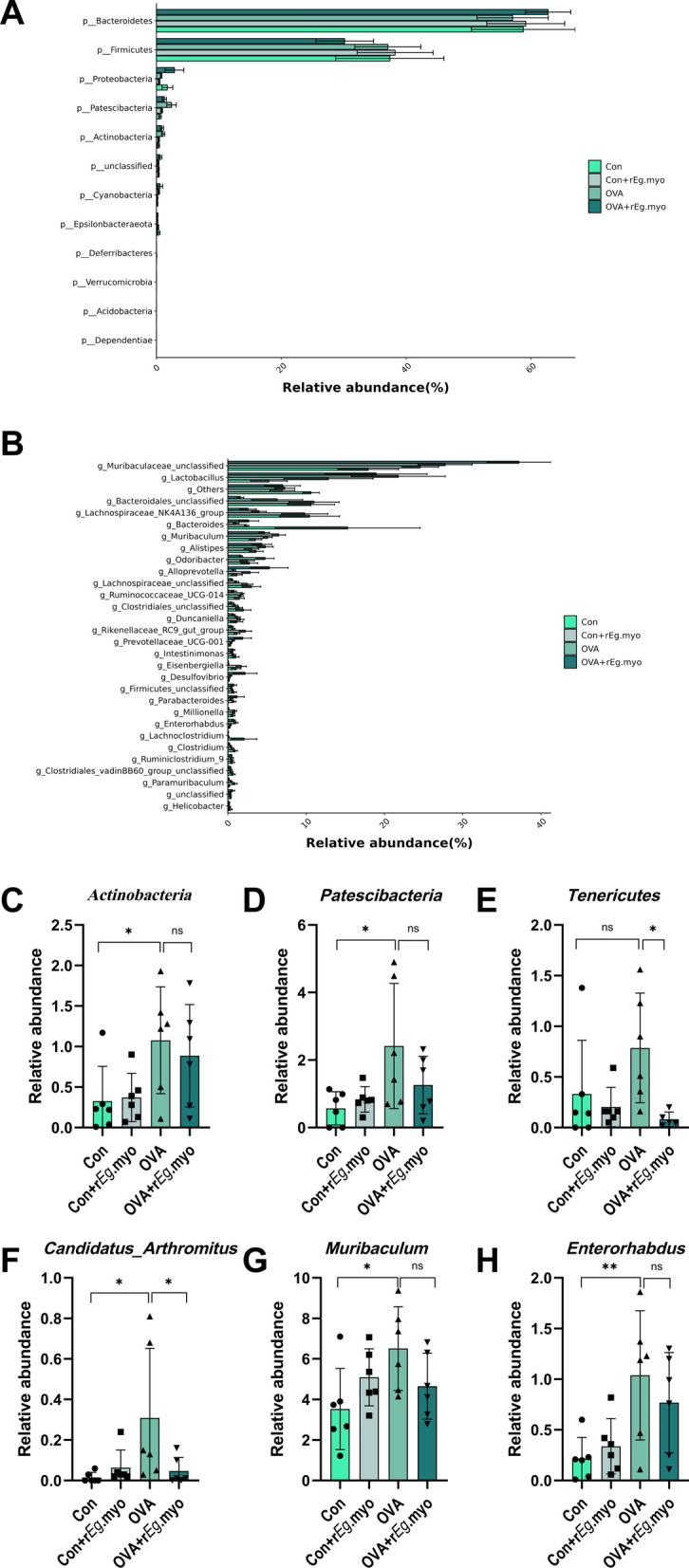
Changes in species abundance of enterobacterial microbiota at the phylum and genus levels. **A** Top 30 species at the phylum level; **B** top 30 species at the genus level; **C**–**H** differential analysis in the abundance of **C** *Actinobacteriota*, **D** *Patescibacteria*, **E** *Tenericutes*, **F** *Candidatus_Arthromitus*, **G** *Muribaculum*, **H** *Enterorhabdus* among the four groups; *n* = 6; * *p* < 0.05, ** *p* < 0.01

Next, we further investigated changes in the composition and abundance of intestinal microbiota at both the phylum and genus levels. We screened the top 30 most abundant phyla in the gut microbiota across all four mouse groups (Fig. 5A). Compared with the Con group, the OVA group showed higher relative abundances of *Actinobacteria*, *Patescibacteria*, and *Tenericutes*. These same phyla showed lower abundances in the OVA+r*Eg*.myo group than in the OVA group (Fig. 5C–E). This trend continued at the genus level. Among the top 30 most abundant genera (Fig. 5B), *Candidatus_ Arthromitus*, *Muribaculum*, and *Enterorhabdus* were significantly upregulated in the OVA group compared with the Con group, but downregulated in the OVA+r*Eg*.myo group compared with the OVA group (Fig. 5F–H).

### r*Eg*.myophilin alters intestinal metabolites in mice with allergic asthma

Based on RNA sequencing of mouse intestinal microbiota, we performed non-targeted metabolomics of mouse feces using high-resolution mass spectrometry. Quality control of the extracted material was ensured through XCMS processing. In both anion and cation modes, the total ion chromatograms showed clear separation of all metabolites with good sample-to-sample fitting curves, indicating the reliability of subsequent data analysis (Additional file 1: Fig. S1A–B).

**Fig. 6:**
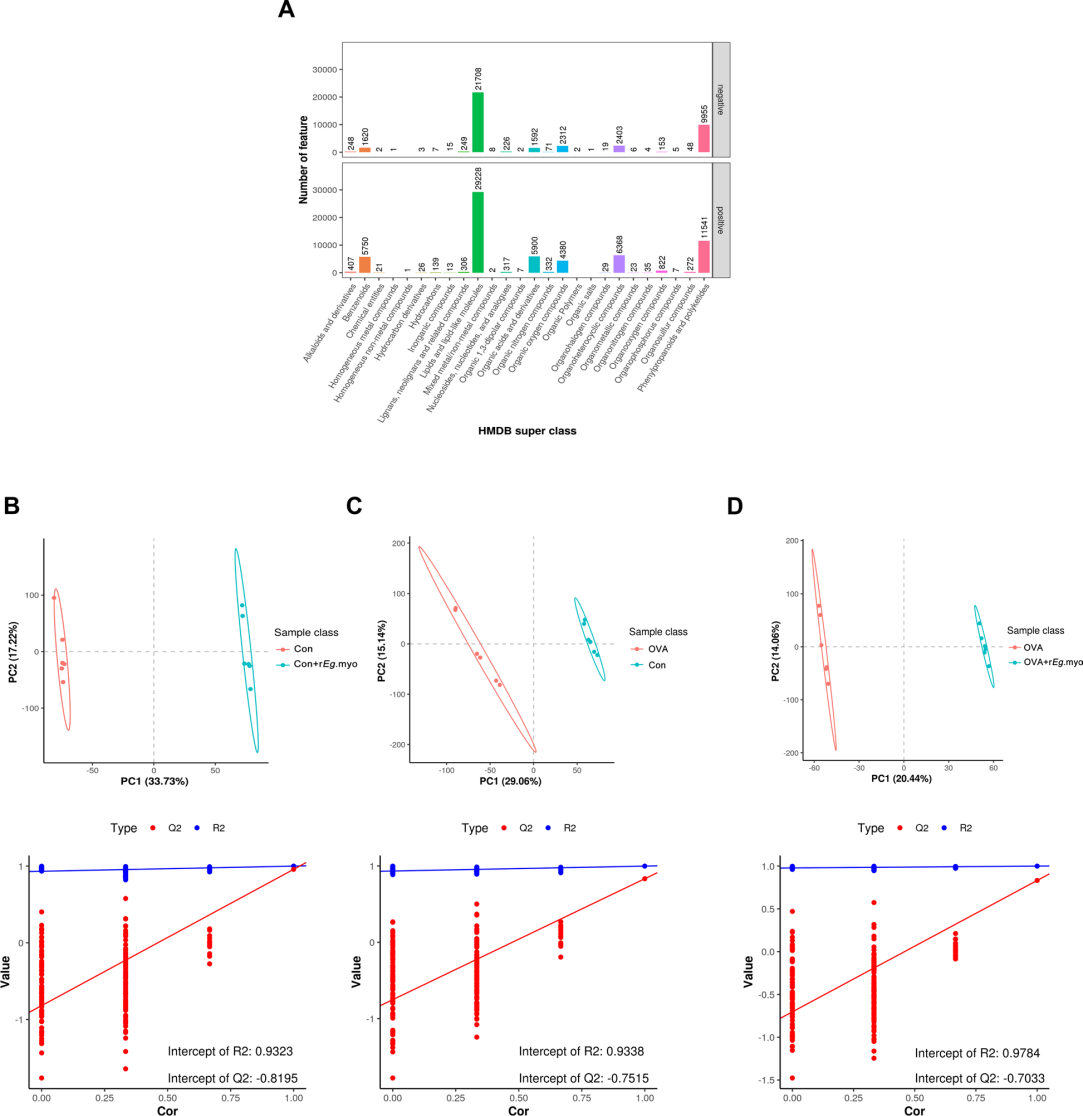
Metabolomic mass spectrometry analysis of mouse fecal samples. **A** Level 1 metabolite annotation based on the Human Metabolome Database. **B**–**D** Differential metabolites identified by partial least squares discriminant analysis between **B** Con group and Con+r*Eg*.myo groups, **C** Con group and OVA groups, and **D** OVA group and OVA+r*Eg*.myo groups

Next, we used the Human Metabolome Database to annotate metabolite ions for both anion and cation modes with high confidence (level 1). Lipids and related compounds were the most abundant class (Fig. 6A). Subsequently, we analyzed group differences using supervised differential discriminant analysis (PLS-DA). Quality control of differential metabolite analysis showed no overfitting (Q2 < 0 across 200 permutations), indicating the model's reliability. The results revealed significant differences between the Con and OVA groups, the Con and Con+r*Eg*.myo groups, and the OVA and OVA+r*Eg*.myo groups (Fig. 6B–D).

### r*Eg*.myophilin corrects the imbalance of gut metabolites in mice with allergic asthma

**Fig. 7:**
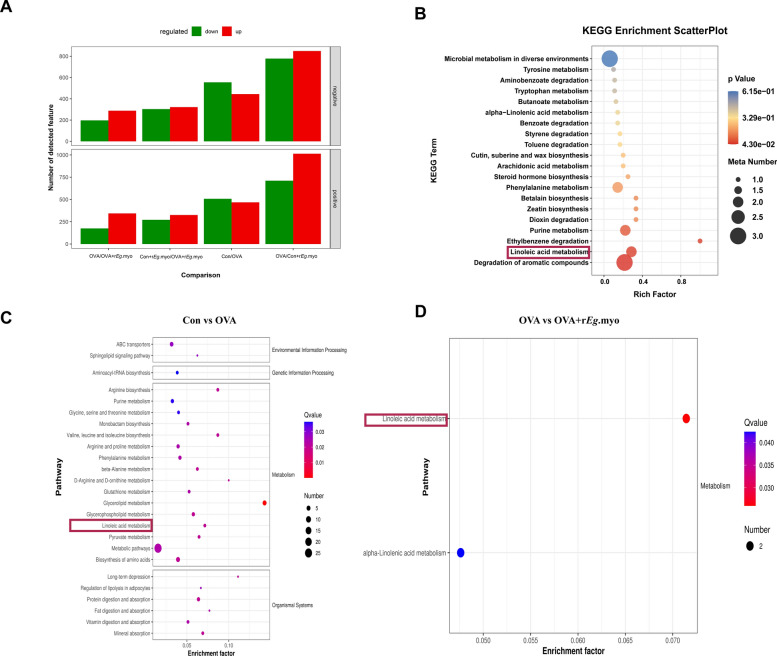
Kyoto Encyclopedia of Genes and Genomes (KEGG) functional enrichment of differential metabolites between groups. **A** Differential metabolite statistics between groups with differential ions satisfying both screening conditions *ratio* ≥ 2 and *ratio* ≤ 1/2; *q* value < 0.05 and *VIP* ≥ 1; **B** functional enrichment of differential metabolites; **C** KEGG functional enrichment of differential metabolites between the Con and OVA groups; **D** KEGG functional enrichment of differential metabolites between the OVA and OVA+r*Eg*.myo groups

Following an overall differential analysis, we compared secondary metabolite ions between groups. A total of 3818 metabolite ions differed in two-by-two comparisons of the four groups in cationic mode, whereas 3736 metabolite ions differed in anionic mode (Fig. 7A). In addition, we functionally enriched differential metabolites between the Con and OVA groups and between the OVA and OVA+r*Eg*.myo groups using the KEGG database. Differential metabolites between the Con and OVA groups were mainly enriched in glycerolipid metabolism and the d-arginine, and d-ornithine metabolism, arginine biosynthesis, and linoleic acid metabolism pathways (Fig. 7C). In contrast, differential metabolites between the OVA and OVA+r*Eg*.myo groups were enriched in the linoleic acid metabolism pathway (Fig. 7D). These findings suggest that r*Eg*.myophilin may modulate the intestinal metabolism of mice with OVA-induced allergic asthma through linoleic acid metabolism metabolic pathways. We also screened differential metabolites between the Con, OVA, and OVA+r*Eg*.myo groups for trends and identified 19 regulatory differential metabolites (Additional file 1: Table S1). We then performed KEGG enrichment analysis on these 19 differential metabolites, which revealed primary enrichment in purine metabolism, ethylbenzene degradation, linoleic acid metabolism, and aromatic compound degradation pathways, indicating that r*Eg*.myophilin may regulate allergic asthma through these metabolic pathways (Fig. 7B).

### r*Eg*.myophilin may alleviate allergic asthma by balancing the linoleic acid metabolic pathway

**Fig. 8:**
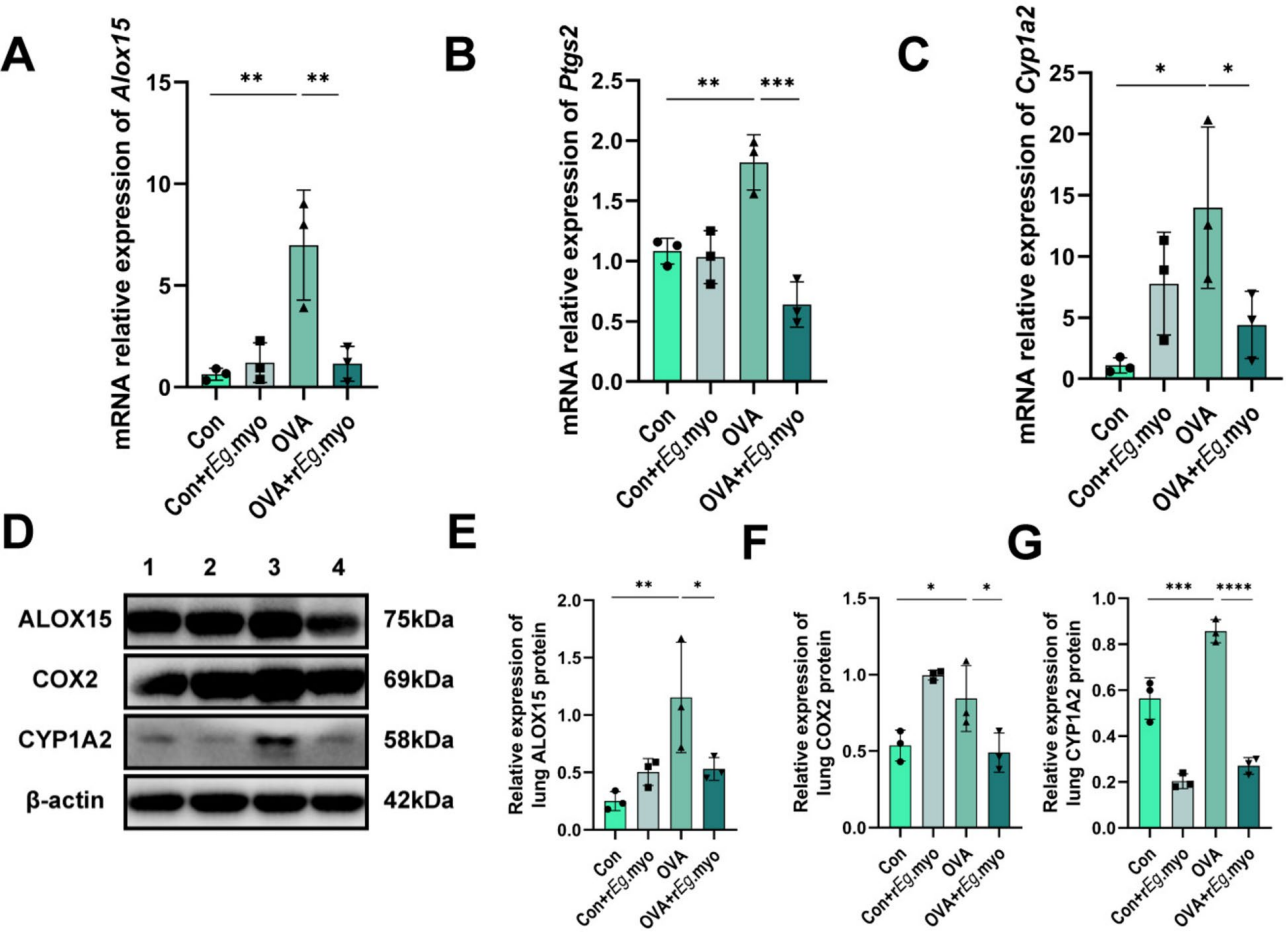
Gene and protein expression of *Alox15* (ALOX15), *Ptgs2* (COX2), and *Cyp1a2* (CYP1A2) in the lung tissues of four mouse groups. **A**–**C** mRNA relative expression of lung **A** *Alox15*, **B** *Ptgs2*, **C** *Cyp1a2*; **D** western blotting strips of ALOX15, COX2, and CYP1A2 (1: Con, 2: Con+r*Eg*.myo, 3: OVA, 4: OVA+r*Eg*.myo). **E**–**G** Protein relative expression of lung **E** ALOX15; **F** COX2; **G** CYP1A2; *n* = 3; * *p* < 0.05, ** *p* < 0.01, *** *p* < 0.001, **** *p* < 0.0001

Functional enrichment analysis showed that differential metabolites were primarily enriched in the linoleic acid metabolic pathway. Linoleic acid is an essential fatty acid in the human body, which is metabolized to arachidonic acid by a series of desaturase and elongase enzymes; therefore, the arachidonic acid metabolic pathway is an important component of the linoleic acid metabolic pathway [19]. Arachidonic acid metabolism predominantly proceeds through the lipoxygenase (LOX), cyclooxygenase (COX), and cytochrome P450 (CYP) pathways. In asthma, ALOX15 in the LOX pathway and COX2 in the COX pathway are significantly upregulated compared with the normal population [20, 21]. Moreover, CYP1A2, a metabolizing enzyme in the CYP metabolic pathway, is a potential target for asthma treatment [22]. Therefore, we analyzed the lung tissues of mice with a focus on the key enzymes of the arachidonic acid metabolic pathway (ALOX15, COX2, and CYP1A2). Quantitative PCR results showed that *Alox15*, *Ptgs2*, and *Cyp1a2* were significantly upregulated in the OVA group relative to the Con group but significantly downregulated in the OVA+r*Eg*.myo group relative to the OVA group (Fig. 8A–C). Western blot results indicated that r*Eg*.myophilin corrected the aberrant upregulation of ALOX15, COX2, and CYP1A2 proteins in the lung tissues of allergic asthma mice (Fig. 8D–G).

### r*Eg*.myophilin exerts immunomodulatory effects in mice with allergic asthma through the joint regulation of intestinal microbiota and metabolites

**Fig. 9:**
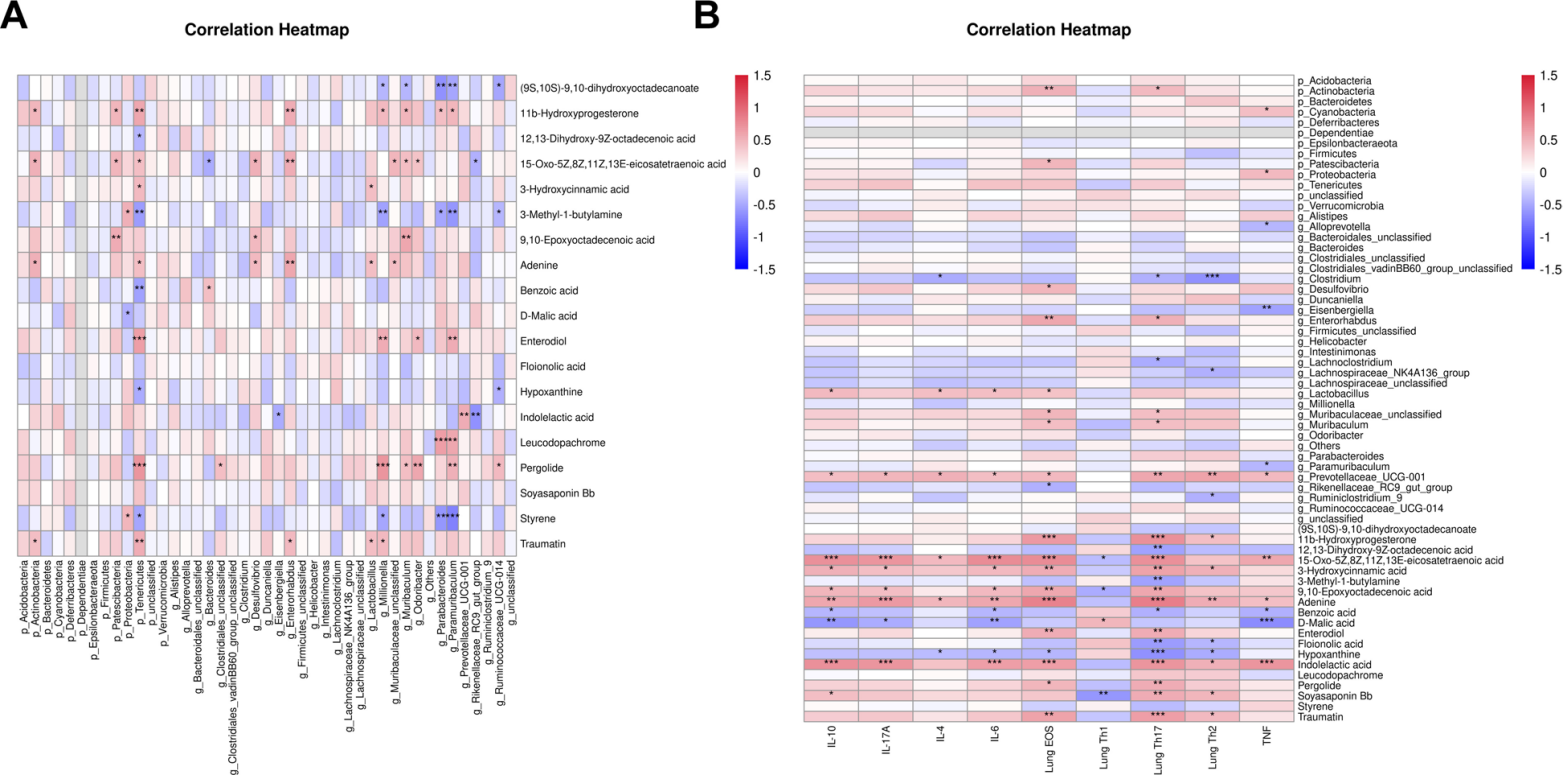
Correlation analysis of immunological indicators, differential microbiotas, and differential metabolites. **A** Correlation analysis of differential microbiotas and differential metabolites. **B** Correlation analysis of immune indicators and differential microbiotas and metabolites. *p < 0.05, **p < 0.01, ***p < 0.001

To further explore the interplay among gut microbiota, metabolites, and immunomodulation in the therapeutic effects of r*Eg*.myophilin on allergic asthma, we performed correlation analysis on the previously identified differential microbiota, metabolites, serum cytokines, and immune cells. Immunological indicators of asthma development, including eosinophils, Th2 cells, Th17 cells, IL-4, IL-6, IL-10, IL-17A, and TNF, showed significant positive correlations with adenine (Fig. 9B). Moreover, adenine was significantly positively correlated with the following bacteria: *Actinobacteria*, *Enterorhabdus*, and *Tenericutes* (Fig. 9A), all of which were significantly upregulated in the OVA group (Fig. 5C, E, and H). Nebulized adenosine increases airway hyperresponsiveness and systemic inflammation in mice with allergic asthma [23]. Thus, these results suggest that r*Eg*.myophilin may jointly regulate intestinal microbiota (*Actinobacteria*, *Enterorhabdus*, and *Tenericutes*), intestinal metabolites (adenine), and immune cells to alleviate OVA-induced airway inflammation in mice with allergic asthma.

## Discussion

In this study, we investigated the mechanisms underlying the role of r*Eg*.myophilin in OVA-induced allergic asthma in mice. First, our results showed that r*Eg*.myophilin effectively alleviated allergic airway inflammation in mice. Subsequent experiments revealed that OVA-induced allergic asthma was characterized by an imbalance in Th1, Th2, and Th17 cells in the lung and serum-associated cytokines, but r*Eg*.myophilin treatment effectively reversed this immune imbalance. Accordingly, we combined mouse gut microbiota sequencing and non-targeted metabolomics analysis of fecal samples to explore the interplay among immunity, gut microbiota, and gut metabolites. Through this three-tiered approach, we elucidated the mechanisms by which r*Eg*.myophilin alleviates allergic asthma in mice, offering new avenues for asthma prevention and treatment.

The human digestive system, a prime site for microorganism colonization, harbors diverse bacterial communities. The hygiene hypothesis proposes that reducing exposure to commensal and pathogenic microorganisms in early childhood may deprive the immune system of adequate stimulation, thus affecting immune system maturation [24]. This phenomenon has been used to explain how gut microbes alleviate airway inflammation by regulating the Th1/Th2 balance [25]. In addition, gut microbes and their associated metabolites can directly or indirectly affect distal organs, including the lungs. The emerging concept of the gut-lung axis suggests a link between chronic lung diseases such as asthma and intestinal symptoms [26]. Conversely, conditions such as ulcerative colitis and Crohn's disease are strongly associated with increased mortality in patients with asthma-associated chronic obstructive pulmonary disease [27]. The intestinal microbiota exerts both pathogenic and therapeutic roles, functioning as a "double-edged sword" to bidirectionally regulate the gut–lung axis. For example, probiotic supplementation with *Bifidobacterium bifidum* increased IL-10 production, thereby protecting mice from lung infections caused by *Klebsiella pneumoniae* [28].

In this study, we first confirmed the mitigating effect of r*Eg*.myophilin on OVA-induced allergic airway inflammation in mice using pathochemical staining and IHC. r*Eg*.myophilin treatment significantly attenuated inflammatory cell infiltration, collagen deposition, and mucus secretion in the lungs of mice. A recent study reported that activated peripheral Th2 cells may be a diagnostic marker for worsening asthma [29]. We then used flow cytometry to examine single-nucleated cells in mouse lungs. Our assay results were consistent with previous reports showing significant upregulation of lung eosinophils, Th2, and Th17 cells as well as downregulation of Th1 cells in the OVA-induced allergic asthma group. r*Eg*.myophilin intervention reversed these trends.

To further elucidate whether r*Eg*.myophilin modulates OVA-induced allergic asthma in mice through gut microbiota-metabolite modulation, we performed 16S rRNA microbiota sequencing and untargeted metabolomics sequencing of mouse fecal samples. Consistent with previous studies, *Actinobacteriota*, *Patescibacteria*, *Candidatus_Arthromitus*, *Muribaculum*, and *Enterorhabdus* all showed increased abundance in the OVA model group; thus, these bacteria may be associated with the development of allergic airway inflammation in mice, suggesting their potential as biomarkers. *Candidatus_Arthromitus*, a conditionally pathogenic bacterium, is significantly more abundant in the gut of patients with gout [30]. r*Eg*.myophilin treatment significantly downregulated *Tenericutes* and *Candidatus_Arthromitus*. Therefore, OVA-induced allergic asthma disrupts the intestinal microbiota in mice, whereas r*Eg*.myophilin intervention significantly modulates the diversity, abundance, and composition of the intestinal microbiota. These findings provide preliminary insights into targeted future use of antibiotics, probiotic administration, and fecal/intestinal microbial transplantation, with differentiated microbiota as a target for treating allergic airway inflammation.

The effects of intestinal microbiota on the human body are mediated by their specific metabolites. Consequently, identifying intestinal bacteria metabolites and their functions represents a growing area of research [31]. In this study, we screened 19 metabolites exhibiting regulatory trends and significant differences among the Con, OVA, and OVA+r*Eg*.myo groups (Additional file 1: Table S1). r*Eg*.myophilin intervention significantly downregulated several metabolites in the OVA group, including pergolide, indoleacetic acid, and 11β-hydroxyprogesteron, but upregulated styrene, benzoic acid, and hypoxanthin. Levels of 3-indoleacetic acid are elevated in children with "early onset asthma," whereas benzoic acid may improve gut function by modulating enzyme activity, redox status, immunity, and microbiota [32, 33]. Thus, r*Eg*.myophilin alters the production and composition of intestinal metabolites in mice with OVA-induced asthma. Moreover, these differential metabolites may represent novel biological targets for improving asthma prevention, diagnosis, treatment, and prognosis. Subsequent functional enrichment analysis of these differentially metabolized compounds showed enrichment predominantly in the linoleic acid metabolic pathway. Linoleic acid is first converted to arachidonic acid in the body. Subsequently, arachidonic acid undergoes metabolism through three pathways: LOX, COX, and CYP. As a member of the LOX family, ALOX15 serves as a crucial enzyme for the oxidation of arachidonic acid. ALOX15 expressed in human airway epithelial cells catalyzes the conversion of arachidonic acid into 15-hydroxyeicosatetraenoic acid, thereby promoting eosinophil migration and goblet cell hyperplasia, which in turn drives the development of airway inflammation [34]. COX2 is a key member of the COX family and a crucial enzyme in arachidonic acid metabolism. Elevated serum COX2 levels in patients with asthma are closely associated with the severity of asthma exacerbation [35]. CYP1A2 is the key enzyme converting arachidonic acid to epoxyeicosatrienoic acids. Reducing CYP1A2 levels can alleviate allergic asthma through the arachidonic acid metabolic pathway [36]. In this study, r*Eg*.myophilin downregulated the levels of ALOX15, COX2, and CYP1A2 in the lung tissue of mice with allergic asthma, thereby exerting an anti-inflammatory effect on the airway.

Intestinal microbiotas are involved in energy absorption, material metabolism, and the immune response, and the stability and resilience of their basic ecological characteristics have important impacts on the human internal environment. Changes in the composition and number of intestinal microbiota and their metabolites affect the metabolism, immune response, and intestinal barrier function of the host [37]. Based on the concept of the gut-lung axis, we performed Spearman's correlation analyses of immunological indices, differential metabolites, and differential microbiota in the Con, OVA, and OVA+r*Eg*.myo groups. *Candidatus Arthromitus* showed a significant increase in the OVA group (Fig. 5F). Although not directly pathogenic itself, *Candidatus Arthromitus* alters the immune environment and has a significant impact on mouse models of inflammatory bowel disease, rheumatoid arthritis, and multiple sclerosis [38, 39].

In summary, r*Eg*.myophilin alleviates airway inflammation in mice with allergic asthma through the lung immune-gut microbiota-gut metabolite axis. However, additional gut microbiota depletion experiments and fecal microbiota transplantation are required to further validate these results. Moreover, our hypothesis that r*Eg*.myophilin acts through the linoleic acid metabolic pathway requires validation using inhibitors or agonists for certain key enzymes. Finally, future research should investigate gut microbiota and metabolites in conjunction with immune cells and molecules.

## Conclusions

Experiments on a mouse model of OVA-induced allergic asthma revealed that r*Eg*.myophilin has a significant mitigating effect on allergic airway inflammation. Specifically, r*Eg*.myophilin improved lung tissue inflammation by reducing infiltration, collagen deposition, and mucus secretion, modulated immune cell populations by increasing Th1 levels and decreasing eosinophil, Th2, and Th17 levels, and induced corresponding changes in cytokine levels. We hypothesize that r*Eg*.myophilin influences the immune response by jointly regulating intestinal microbiota and metabolites, primarily through the linoleic acid metabolic pathway, thereby alleviating allergic airway inflammation. In addition to highlighting novel biomarkers for asthma prevention and treatment, this research provides theoretical support for r*Eg*.myophilin as a potential therapeutic against allergic asthma.

## Supplementary Information

Additional file 1.

## Data Availability

Data supporting the main conclusions of this study are included in the manuscript.

## Abbreviations

IgE: Immunoglobulin E
OVA: Ovalbumin
r*Eg*.myo: Recombinant *echinococcus granulosus* myophilin
TNF: Tumor necrosis factor
PBS: Phosphate-buffered saline
IL: Interleukin
KEGG: Kyoto encyclopedia of genes and genomes
Th cells: T helper cells
ALOX15: Arachidonate 15-lipoxygenase
CYP1A2: Cytochrome P450 1A2
COX2: Cyclooxygenase-2
