# Supplementary material for: Recombinant Echinococcus granulosus myophilin alleviates airway inflammation in mice with ovalbumin-induced allergic asthma via the gut microbiota-metabolite-immune axis
Source: Parasit Vectors. 2026 May 19;19:288. doi: 10.1186/s13071-026-07444-8 (PMC13361099; doi:10.1186/s13071-026-07444-8)
Supplement: Supplementary file 1 — Additional file 1. [file 13071_2026_7444_MOESM1_ESM.docx]

Supplementary Material

**Recombinant *Echinococcus granulosus* myophilin alleviates airway inflammation in mice with ovalbumin-induced allergic asthma via the gut microbiota-metabolite-immune axis**

**Zhichao Zhou ^#^, Leiji Fu ^#^, Jianwen Wu ^#^, Rou Wen, Zexin Dang, Junyou Wu, Xiaomin Zhang, Sijia Bao, Wenxuan Li, Xiaoping Gao , Mei Yin *, and Jiaqing Zhao***

*** Correspondence:**

Jiaqing Zhao^*^  zhaojq@nxmu.edu.cn

Mei Yin^*^ [Yinm@nxmu.edu.cn](mailto:Yinm@nxmu.edu.cn)

^#^These authors contributed equally to this work

# Supplementary Figures


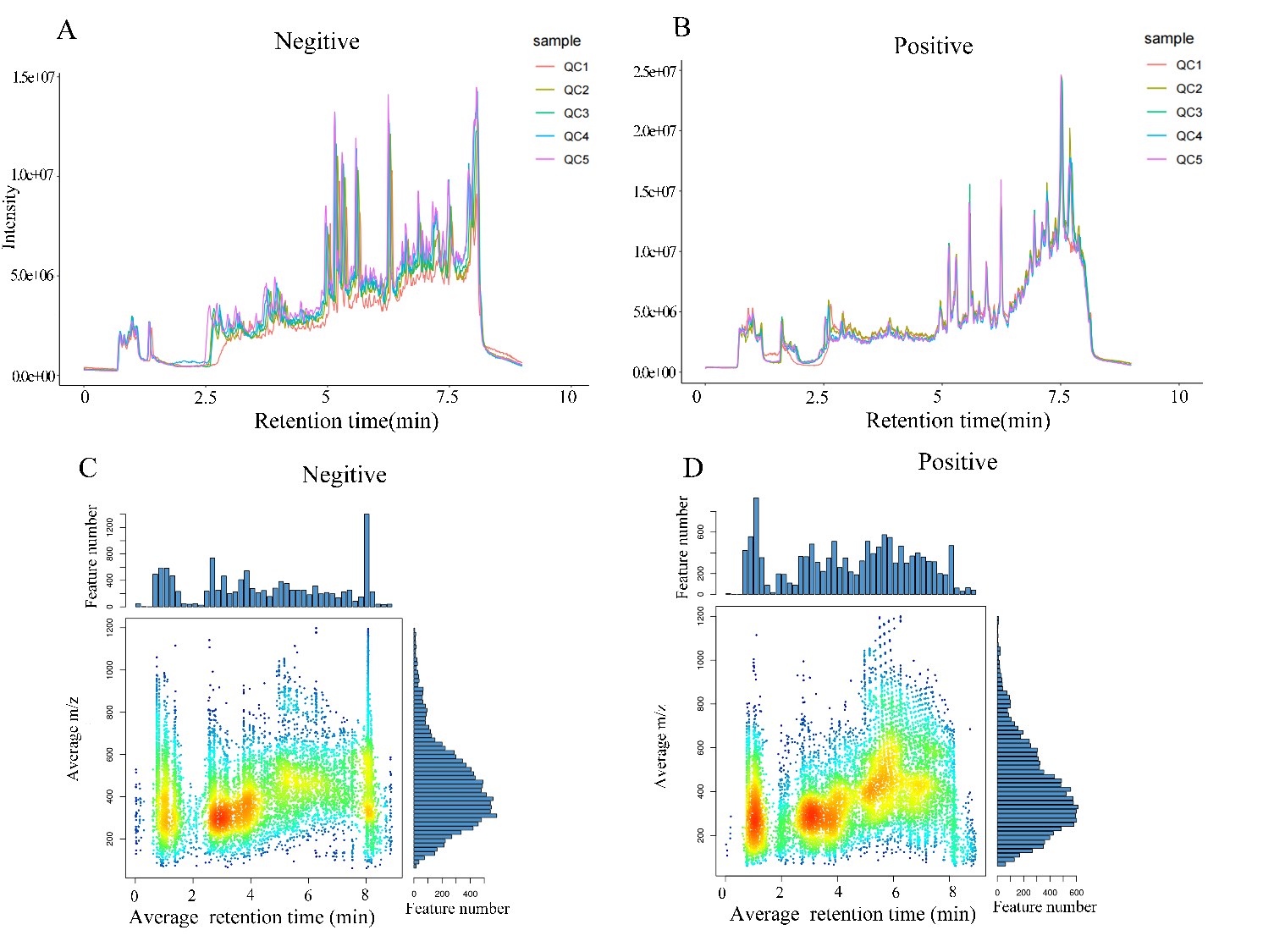


**Supplementary Figures 1. Quality Control for Metabolomic Mass Spectrometry Analysis of Mouse Feces**

**A.** **Quality control in negative ion mode; B.** **Quality control in positive ion mode.**

# Supplementary Table

| MS2Metabolite | P value | | Ratio | | VIP | | Trend | | MZ | RT(min) |
| --- | --- | --- | --- | --- | --- | --- | --- | --- | --- | --- |
|  | Con vs OVA | OVA vs OVA+r*Eg*.myo | Con vs OVA | OVA vs OVA+r*Eg*.myo | Con vs OVA | OVA vs OVA+r*Eg*.myo | Con vs OVA | OVA vs OVA+r*Eg*.myo |  |  |
| 15-Oxo-5Z,8Z,11Z,13E-eicosatetraenoic acid | 0.0000 | 0.0000 | 0.2568 | 3.0703 | 2.5177 | 2.5562 | down | up | 319.2236 | 6.7121 |
| 9,10-Epoxyoctadecenoic acid | 0.0000 | 0.0000 | 0.2350 | 4.0241 | 2.5962 | 3.0592 | down | up | 297.2419 | 6.7086 |
| Soyasaponin Bb | 0.0002 | 0.0015 | 0.3186 | 2.8894 | 2.1511 | 2.5623 | down | up | 987.5059 | 5.2800 |
| Adenine | 0.0011 | 0.0000 | 0.0820 | 3.0802 | 3.7508 | 1.8238 | down | up | 136.0609 | 1.2074 |
| Enterodiol | 0.0000 | 0.0000 | 0.4007 | 2.1772 | 2.0202 | 2.1045 | down | up | 301.1401 | 4.4319 |
| Traumatin | 0.0001 | 0.0000 | 0.3785 | 2.3377 | 2.1810 | 2.3110 | down | up | 211.1304 | 4.8528 |
| Pergolide | 0.0001 | 0.0000 | 0.4347 | 2.5114 | 1.9336 | 2.5931 | down | up | 315.1926 | 6.3507 |
| Indolelactic acid | 0.0001 | 0.0004 | 0.3956 | 2.1000 | 1.5726 | 1.5078 | down | up | 204.0631 | 4.0071 |
| 3-Hydroxycinnamic acid | 0.0001 | 0.0000 | 0.4336 | 2.1392 | 1.7863 | 1.9793 | down | up | 165.0538 | 3.1286 |
| 11b-Hydroxyprogesterone | 0.0008 | 0.0002 | 0.2849 | 2.1089 | 2.6141 | 1.8186 | down | up | 331.1864 | 6.0137 |
| Leucodopachrome | 0.0005 | 0.0029 | 0.1628 | 3.9219 | 2.2106 | 2.0153 | down | up | 194.0455 | 2.6029 |
| D-Malic acid | 0.0058 | 0.0001 | 2.1591 | 0.2049 | 1.2299 | 2.8985 | up | down | 133.0115 | 1.1036 |
| (9S,10S)-9,10-dihydroxyoctadecanoate | 0.0049 | 0.0000 | 3.4920 | 0.3393 | 1.9497 | 2.3513 | up | down | 315.2493 | 5.6521 |
| Floionolic acid | 0.0004 | 0.0000 | 2.4018 | 0.3629 | 1.8413 | 2.6908 | up | down | 331.2439 | 5.0235 |
| Styrene | 0.0006 | 0.0032 | 10.7486 | 0.1095 | 2.7974 | 2.9777 | up | down | 105.0693 | 2.9751 |
| Benzoic acid | 0.0000 | 0.0000 | 2.4990 | 0.1923 | 1.4616 | 3.2352 | up | down | 123.0431 | 3.7313 |
| Hypoxanthine | 0.0010 | 0.0025 | 2.3745 | 0.4051 | 1.7256 | 2.1260 | up | down | 137.0450 | 1.8788 |
| 12,13-Dihydroxy-9Z-octadecenoic acid | 0.0004 | 0.0000 | 2.1652 | 0.2952 | 1.4863 | 2.9107 | up | down | 315.2522 | 5.0039 |
| 3-Methyl-1-butylamine | 0.0003 | 0.0011 | 10.2589 | 0.1410 | 3.2560 | 3.2510 | up | down | 88.1115 | 2.7272 |

**Supplementary table 1.** **Statistical table of differential metabolites**
